# Nosebleed: With Clinical Reflections

**Published:** 1884-03

**Authors:** Ad. Lippe

**Affiliations:** Philadelphia


### NOSEBLEED: WITH CLINICAL REFLECTIONS.

AD. LIPPE, M. D., PHILADELPHIA.

A married lady, age fifty years, on whom we had waited for over thirty years, had inherited the predisposition to tuberculosis and had all the premonitory symptoms some thirty years ago but by care and careful treatment enjoyed comparatively good health. On the 12th of January she consulted me about some new harassing symptoms. There had been a formation of increasing scabs in her nostrils; they first appeared on the left side and now extended to right side. When she blew her nose they came away and then came a few drops of blood; the nostrils felt sore. She took that evening one dose of Lachesis$^{cm}$ (F. C.). When she awoke early next morning her nose began to bleed profusely; the blood would coagulate for a short time in the nostrils and in the posterior nares, but she was impelled to blow her nose and hawk up this coagulated blood, falling from the posterior nares into the throat. She became quite alarmed at this apparent hæmorrhage and requested me to call and see her. *We took in the situation,* and came to the conclusion that this alarming condition was caused by that dose of Lachesis and *thus,* of course, *we waited.* The hæmorrhage ceased before noon entirely, the subsequently formed scabs in the nostrils became smaller and smaller; the lady has not needed any further medication, as she found the healing process was progressing.

*Comments.*—As there *never* will be found a specific for nosebleed or for any other diseased condition, it behooves us in every individual case *to take in the situation* and individualize. In this case it was apparent that the violent nosebleed was caused by the one dose of Lachesis given the evening before for "*the totality of symptoms.*" We administered a well-proven remedy in an appropriate dose and the result demonstrated the correctness of our supposition.

As we live in an age in which labor-saving machines are at a premium, it is not to be wondered that there exist medical practitioners who, affected by the prevailing epidemic, exert their ingenuity to discover medical-saving machines, and to what spot on the globe can we look more hopefully for an inventor of new medical principles, new laws, and labor-saving machines, than "Gotham," the Eldorado of medical "cranks"? One of the shining lights has advertised that *he* has found a specific for nosebleed which can be obtained from him in a highly potentized form—which makes up for the want of a proving of said specific nosebleed cure. This method would save labor if successful, but as it cannot be done we will undertake to give a labor-saving analysis of the various remedies good for nosebleed under *certain conditions.*

NOSEBLEED IN GENERAL: Aconite, Agar., Alum., Ambr., Amm. carb., Amm. caust., Anac., Ang., Ant. cr., Asterias rub., Argent., Arnica, Arsen., Asaf., Baryta, Bellad., Borax, Bovist., Brom., Bryon., Cactus gr., Calc. c., Cannab., Canth., Caps., Carbo an., Carbo veg., Caust., China, Chinin., Cham., Cina, Coff., Colch., Con., Corall., Croc., Crotalus, Cupr. ac., Diad., Digit., Dros., Dulc., Euphr., Ferr., Granat., Graph., Gumi gutti., Hep., Hyos., Igt., Indigo, Iod., Ipec. Hamamelis, Kali c., Kali chlor., Kali hydroj., Kreasot., Lach., Led., Lyc., Magn. carb., Magn. m., Magn. sulph., Meny., Mephit., Merc. v., Merc. corr., Merc. cyan., Mercurialis, Millef., Mosch., Mur. ac., Natr. carb., Natr. mur., Natr. sulf., Nitr., Nitr. ac., Nux v., Paris, Petro., Peruvian bals., Phosph., Phosph. ac., Puls., Ratanh., Rhod., Rhus, Ruta, Sabad., Sabina, Sarsap., Secale, Senega, Sep., Sil., Spong., Stann., Stront., Sulph., Sulph. ac., Tarax., Tart. em., Tereb., Thuja, Veratr., Vinca, Viol. od.

FROM ONE NOSTRIL: Crot., Corall. rubr.
— the right nostril: Gummi gutti., Sars., Veratr.
— the left nostril: Ferr. ac., Nitr., Merc. sol., Rhodod.

Blood, dark : Lach., Nitr. ac., Puls., Sulf. ac.
— fluid, thin : Kreosot., Crotalus.
— pale : Baryta, Carbo an., Crotal., Digit., Dulc., Kreosot., Hyos., Led., Sabad.
— black : Crocus., Nitr. ac., Kreosot.
— sharp, like vinegar : Nitr., Sil.
— warm : Dulc.
— thick (heavy) : Croc., Kreosot., Lach.
— clots, forming : Crot., Merc., Lach., Nitr. ac.

CONDITIONS.

In the Morning : Agnu., Ambr., Amm. c., Bell., Berb., Borax, Bov., Bry., Calc. c., Canth., Caps., Carbo an., Carbo veg., China, Colch., Dros., Graph., Hepar, Kali c., Kreosot., Lach., Magnesia, Meny., Natr., Nitr. ac., Nux v., Petr., Phosph., Rhus, Sep., Stann., Sulph., Thuja.
— — — while in bed : Baryt., Bry., Caps., Carbo veg., Magnesia, Stann.
— — — when awaking : Stann.
— — — when rising : Stann.
— — — every morning : Kali c.
— — — better : Magn. mur.
In the Forenoon : Carbo veg.
At Noon, before eating : Tarax.
In the Afternoon : Carbo a., Lyc., Natr. sulph., Nitr., Sulph., Tart. em.
Every Evening : Ant. crud.
In the Evening : Ant. cr., Borax, Coff., Colch., Dros., Ferr., Graph., Lach., Lyc., Phosph., Sepia, Sulph., Sulph. ac.
During Sleep : Bry., Merc. corr., Merc. v., Natr. s., Nitr. ac., Puls., Sulph., Veratr.
At Night : Bell., Calc., Carb. veg., Corall, Graph., Kali chlor., Magn. m., Magn. sulph., Natr. m., Natr. sulph., Rhus, Verat.
Coughing, when : Bell., Bry., Carbo an., Carbo v., Dros., Ferr., Hyos., Merc., Natr. m., Nitr. ac., Puls., Sulph.
Eating, after : Ammon. c., Argent.
Sneezing, when : Con., Magn. c.
Hawking, when : Rhus.
Congestion to the head, with : Acon., Alum, Bellad., China, Con., Crot., Graph., Rhus.
Chill and heat, between : Eupat. perf.
Giddiness, after : Carbo an.
Singing, after : Hepar.
Sitting, while : Carbo an., Sulph. ac.

WALKING, when in the open air : Lyc., Natr. c.
STOOL, while straining to : Carbo v., Phosph.
— during: Phosph.
WASHING, while: Amm. c., Dros.
STANDING, while : Sulph. ac.
STOOPING, while: Dros., Ferr., Natr. m., Rhus, Sil.
— after : Carbo veg.
VOMITING, after: Ars.
OVERHEATED, after being: Sep., Thuja.
— during and after the use of spirituous liquors : Acon., Bell., Bry., Lachesis, Nux.
MENSTRUATION, during: Natr. c.
— before: Lach.
— being suppressed : Bry.
— being too scanty : Puls., Secale, Sep.
— being too profuse: Acon., Calc. c., Croc., Sabina.

CONCOMITANT SYMPTOMS.

Eye and chest symptoms are relieved : Brom.
Morning headache is relieved : Magn. sulf.
Bleeding from all apertures of the body : Crotal., Lach.
Congestions to the head, with : Alum, Graph.
Chest, with pain in the: Carbo veg.
Face, with paleness of the: Carbo veg.
— — heat of the: Graph.
Limbs, with pain in the: Natr. c.
Palpitation of the heart, with : Graph.
Headache, with : Alum, Carbo a., Dulc., Lach., Magn. c.
Fainting, with : Calc. c., Crot.
Giddiness, with: Carbo an., Crotal., Lach.
Perspiration, cold on the forehead: Crotal.
Vanishing of vision : Indigo.
Coryza, with : Ars., Puls.
Palpitation of the heart, with : Cactus grand.

